# Identification and Concentration of Some Flavonoid Components in Malaysian Young Ginger (*Zingiber officinale* Roscoe) Varieties by a High Performance Liquid Chromatography Method

**DOI:** 10.3390/molecules15096231

**Published:** 2010-09-03

**Authors:** Ali Ghasemzadeh, Hawa Z E Jaafar, Asmah Rahmat

**Affiliations:** 1 Department of Crop Science, Faculty of Agriculture, University Putra Malaysia, 43400 UPM Serdang, Selangor, Malaysia; E-Mail: upmali@yahoo.com (A.G.); 2 Department of Nutrition & Dietetics, Faculty of Medicine & Health Sciences, University Putra Malaysia, 43400 UPM Serdang, Selangor, Malaysia; E-Mail: asmah@medic.upm.edu.my (A.R.)

**Keywords:** 1,1-diphenyl-2-picryl-hydrazyl assay, total flavonoids, antioxidant activity, DPPH scavenging activity, Halia Bentong, Halia Bara

## Abstract

Flavonoids make up one of the most pervasive groups of plant phenolics. Due to their importance in plants and human health, it would be useful to have a better understanding of flavonoid concentration and biological activities that could indicate their potentials as therapeutic agents, and also for predicting and controlling the quality of medicinal herbs. Ginger (*Zingiber officinale* Roscoe) is a famous and widely used herb, especially in Asia, that contains several interesting bioactive constituents and possesses health promoting properties. In this study, total flavonoids and some flavonoid components including quercetin, rutin, catechin, epicatechin, kaempferol and naringenin were extracted from the leaves and rhizomes of two varieties of *Zingiber officinale* (Halia Bentong and Halia Bara) at three different growth points (8, 12 and 16 weeks after planting), and analyzed by a high performance liquid chromatography (HPLC) method in order to determine the potential of the subterranean part of the young ginger. The results showed that Halia Bara had a higher content of flavonoids in the leaves and rhizomes as compared to Halia Bentong. In both varieties, the concentration of flavonoids in the leaves decreased (Halia Bentong, 42.3%; Halia Bara 36.7%), and in the rhizomes it increased (Halia Bentong 59.6%; Halia Bara 60.1%) as the growth period increased. Quercetin was abundant in both varieties. The antioxidant activity determined by the 1,1-diphenyl-2-picryl-hydrazyl (DPPH) assay showed high activities (65.7%) in the leaves of Halia Bara at 8 weeks after planting. Results suggested a good flavonoid content and antioxidant activity potential in ginger leaves at 8 weeks after planting. The leaves of these ginger varieties could be useful for both food flavourings and in traditional medicine.

## 1. Introduction 

Plants and herbs consumed by humans may contain thousands of different phenolic acid and flavonoid components. The effect of dietary phenolics is of great current interest due to their antioxidative and possible anticarcinogenic activities [1]. Phenolic acids and flavonoids also function as reducing agents, free radical scavengers, and quenchers of singlet oxygen formation [2]. Antioxidant compounds that scavenge free radicals help protect against degenerative diseases [3]. Phenolic components play important roles in the control of cancer and other human diseases. For example, ginger has long been used in traditional medicine as a cure for some ailments including inflammatory diseases [4]. It was found that flavonoids reduce blood-lipid and glucose, and enhance human immunity [5]. Flavonoids are also a kind of natural antioxidant substances capable of scavenging free superoxide radicals, thus displaying anti-aging properties and reducing the risk of cancer. At present, flavonoids are extracted, among other sources, from ginkgo leaves [6], kudzu root [7], lotus leaves [8] and ginger rhizomes and leaves [9]. Ginger is an important horticultural crop in tropical Southeast Asia. It produces a pungent, aromatic and bioactive rhizome that is valued all over the world either as a spice or herbal medicine. Ginger is known as a resource with high phenolic contents, wide availability and low price [10], and therefore, it can serve as a cheap and important food material. Ginger is a natural food component with many active phenolic compounds such as gingerol and shagaol, and it has been shown to have anti-cancer and antioxidant effects [11]. Gingerol may reduce nausea caused by motion sickness or pregnancy and may also relieve migraines [12].

Light is known to regulate plant growth and development, and also the biosynthesis of both the primary and secondary metabolites [13,14]. Apart from their role to benefit health, antioxidants are added to foods to prevent or delay food oxidation initiated by free radicals formed during their exposure to some environmental factors such as air, light and temperature. At present most of the antioxidants in use are manufactured synthetically. In Asia, rhizomes of ginger varieties (family Zingiberaceae) have been widely used as spices in food or condiments [15]. The rhizomes are usually eaten raw or cooked as vegetables and used for flavouring foods. Traditionally, leaves of *Elettariopsis latiflora* (family Zingiberaceae) have been used to relieve flatulence, to improve appetite and as an antidote to poisons [16]. In Japan, leaves of *Alpinia zerumbet* are sold after drying as a herbal tea, and are commonly used to flavour noodles and to wrap rice cakes in celebrations. In another study the diuretic and anti-ulcerogenic properties of *A. zerumbet* leaves have been reported [17].

In Malaysia, only ginger rhizomes are consumed as a food flavoring and the leaves are discarded. Information on the flavonoid contents of plant foods and plant parts commonly consumed in Malaysia are still scarce. Such data would be useful to provide information on foods containing high levels of beneficial components. In the present study, we identified some of important phenolic components (flavonoids) in both of the leaves and rhizomes of two varieties of Malaysian ginger, and the antioxidant activities in these varieties was considered. The variation of flavonoid concentration as a function of growth period of young ginger was also evaluated. 

## 2. Results and Discussion

### 2.1. Total flavonoids (TF)

From the data presented in Table 1, it is apparent that TF content was high in the leaves of both varieties. High levels of TF and total phenolic (TP) contents in the leaves in comparison with other parts of medicinal plants have been reported in previous studies. TF content in leaves of *Psidium guajava* were higher than in stems [18]. High levels of TP were reported in the leaves (291 mg/100 g fresh weight) of *Zingiber officinale* compared to the rhizomes (157 mg/100 g fresh weight) [4]. Leaf chloroplasts have the capability to localize phenolic compounds, some of which are specific only to these organelles [19]. It is also apparent from Table 1 that the TF contents decreased in the leaves and stems of ginger varieties from 8 to 16 weeks after planting, and *vice versa*, increased in the rhizomes. Comparison of the TF results between ginger (5.54–11.14 mg/g DW) and other plants, for example onion leaves (1.54 mg/g DW), semambu leaves (2.041 mg/g DW), black tea (1.41 mg/g DW), papaya (1.26 mg/g DW), bird chilli (1.66 mg/g DW), garlic (1.2 mg/g DW) and guava (1.12 mg/g DW) [20], shows the good potential of this component in these ginger varieties. 

### 2.2. HPLC analysis results 

**Figure 1:**
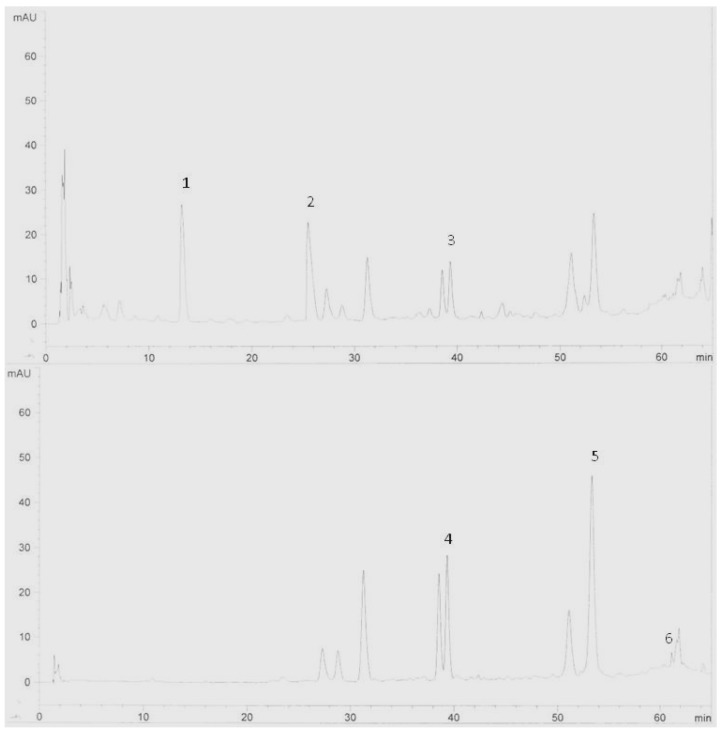
HPLC chromatogram of *Zingiber* *officinale* variety Halia Bara extracts (leaves). Identification of compounds: catechin (1), epicatechin (2), naringenin (3), rutin (4), quercetin (5) and kaempferol (6).

**Figure 2:**
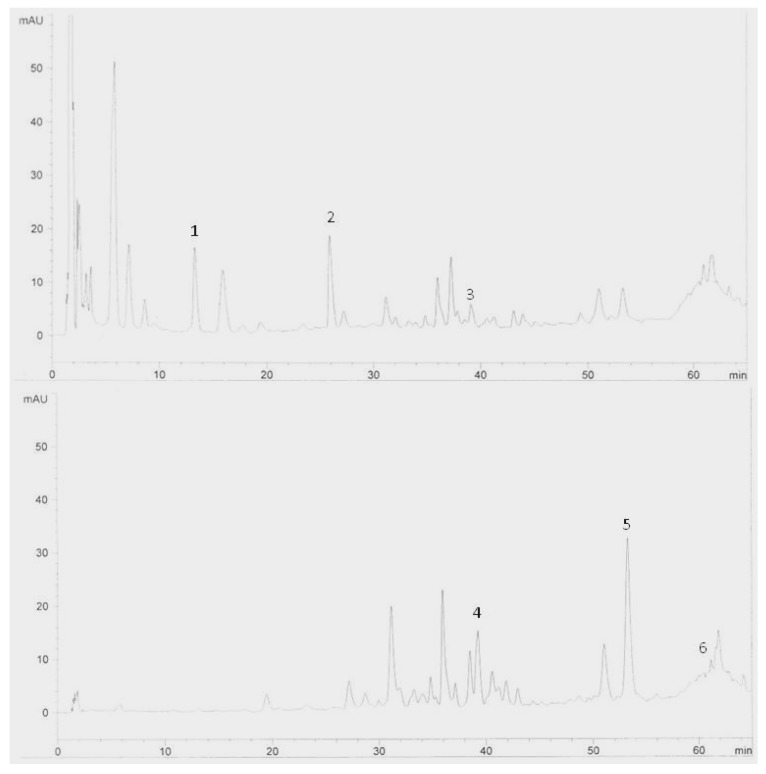
HPLC chromatogram of Zingiber officinale variety Halia Bara extracts (rhizomes). Identification of compounds: catechin (1), epicatechin (2), naringenin (3), rutin (4), quercetin (5) and kaempferol (6).

The results obtained from the preliminary analysis of flavonoids are listed in Table 1. It was shown that Halia Bara had more flavonoids content in the leaves and rhizomes compared to Halia Bentong. What is interesting in this data is that in both varieties the concentration of flavonoids in the leaves decreased as the growth period increased, but in the rhizome it increased with increasing growth period. The decreases in Halia Bentong leaves were 10.0% (quercetin), 64.5% (rutin), 19.7% (catechin), 18.0% (epicatechin), 9.3% (naringenin) and 15.7% for kaempferol. In Halia Bara, they were 24.8% (quercetin), 2.9% (rutin), 24.4% (catechin), 42.2% (epicatechin), 2.5% (naringenin) and 21.2% for kaempferol. In the rhizome, the increases of these components in Halia Bentong were 37.5% (quercetin), 29.0% (rutin), 20.0% (catechin), 36.4% (epicatechin), 32.7% (naringenin) and 48.9% for kaempferol. In Halia Bara they were 25.5% (quercetin), 45.5% (catechin), 78.0% (epicatechin), 25.0% (naringenin) and 20.0% for kaempferol. The HPLC chromatograms from the extracts of the leaves (Figure 1) and the rhizomes (Figure 2) show some of the flavonoid compounds found in Halia Bara. The decrease of TF in Halia Bentong leaves was 42.3%, and in Halia Bara leaves it was 36.7%. The increase in TF content in Halia Bentong rhizomes was 59.6% and in Halia Bara it was 60.2%. In the family Zingiberaceae and especially *Zingiber officinale*, it is generally believed that secondary metabolites produced by the plants are transported to the rhizomes where they are then accumulated [21,22,23]. Najda *et al.* [24] have reported increases in total flavonoids in caraway roots during the vegetative growth period. This may imply that rhizomes of *Z. officinale* have higher stored amounts of flavonoids than other plant parts. 

Chan *et al*. [4] reported that leaves in ginger with high levels of TF and TP had higher antioxidant activities than rhizomes. The results of previous studies [25,26,27,28] showed that some of the flavonoid components such as quercetin and catechin had anticancer activities, and these components were able to inhibit cancer cell growth. The anticancer activities of ginger were also substantiated [29]. Results of the current research (Table 2) showed that flavonoids are important components of this plant, and that some of its pharmacological effects could be attributed to the presence of valuable TF and TP constituents. The quercetin content in the leaves and rhizomes of Halia Bara showed higher values compared to some plants, for example red chilli (0.799 mg/g DW), bird chilli (0.392 mg/g DW) and bell pepper (0.448 mg/g DW), and comparable to black tea (1.107 mg/g DW), onion (1.49 mg/g DW) and semambu (1.18 mg/g DW) [19]. A high content of quercetin (1.29 mg/g DW) was obtained in the leaves of Halia Bara at 16 weeks after planting. During the growth period variable contents of rutin and naringenin were found in the leaves and rhizomes of both varieties. Catechin and epicatechin contents in both varieties were high in the leaves at 8 weeks after planting. High content of catechin and epicatechin were detected from Halia Bara leaves (0.56 mg/ g DW; 0.19 mg/g DW) at 8 weeks after planting. Kaempferol is a rare flavonoid in plants. In Halia Bara and Halia Bentong, however, it was detected in the leaves and rhizomes in low concentrations. However, compared with green chilli (0.039 mg/g DW), sengkuang (0.037 mg/g DW), white radish (0.0383 mg/g DW) and pegaga (0.0205 mg/g DW, both ginger varieties recorded high contents (0.023–0.068 mg/g DW) of kaempferol. Ginger varieties, when compared with some plants such as cekur manis (0.323 mg/g DW), pumpkin (0.371 mg/g DW), and carrot (0.140 mg/g DW) [20], showed low contents of kaempferol. A high content of kaempferol (0.068 mg/g DW) was obtained from Halia Bara rhizomes at 16 weeks after planting. 

The results of this study indicate that quercetin is the main flavonoid component in ginger. There has not been any documentation on the flavonoids components of Malaysian ginger varieties, and that our results were the first to indicate the variation of flavonoid concentration in different ginger parts during the growth period. This is an important issue for future research.

### 2.3. DPPH radical scavenging activity

**Figure 3:**
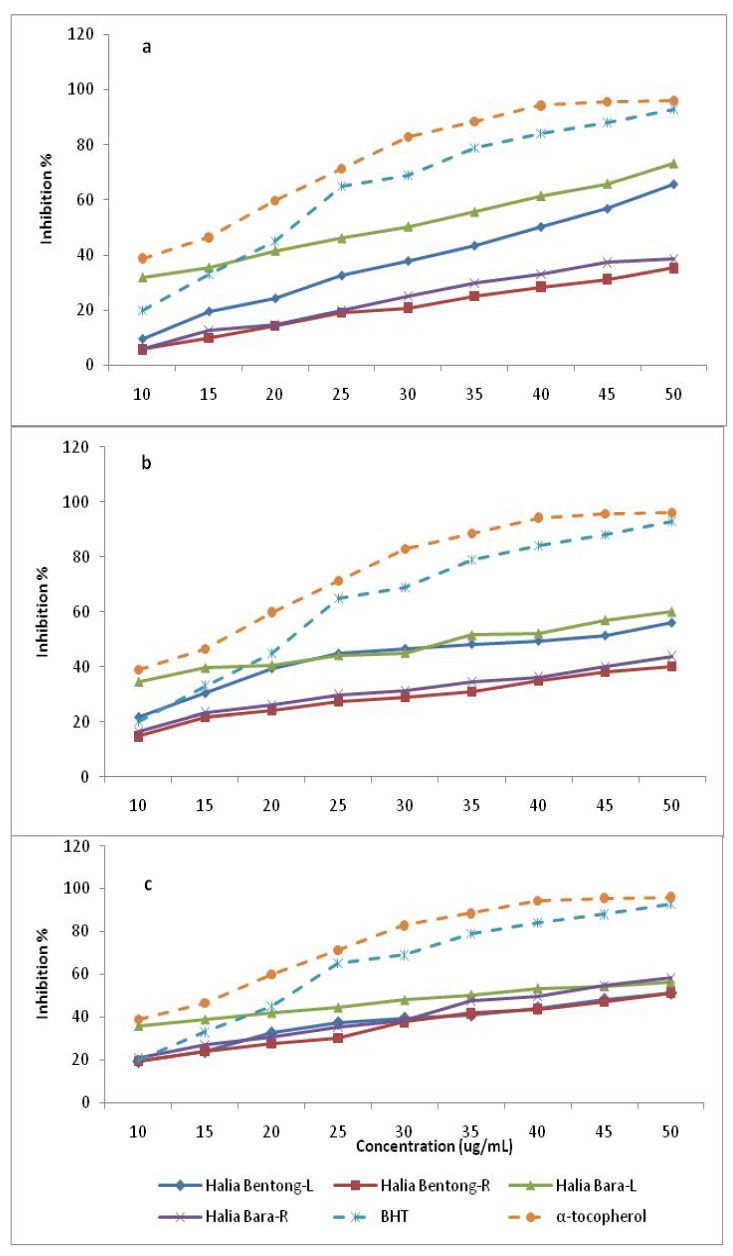
DPPH radical scavenging activity of the methanolic extracts in different parts of two varieties of *Zingiber officinale* during the growth period compared with positive controls, BHT and α-tocopherol. L and R represent leaves and rhizomes of ginger, respectively. a, b and c signify 8, 12 and 16 weeks after planting, respectively.

The dose-response curve of DPPH radical scavenging activities of the methanolic extracts of the leaves and rhizomes in two varieties of *Zingiber officinale* at different harvest times compared with the standardized BHT and α-tocopherol are shown in Table 3 and Figure 3. 

**Figure 4:**
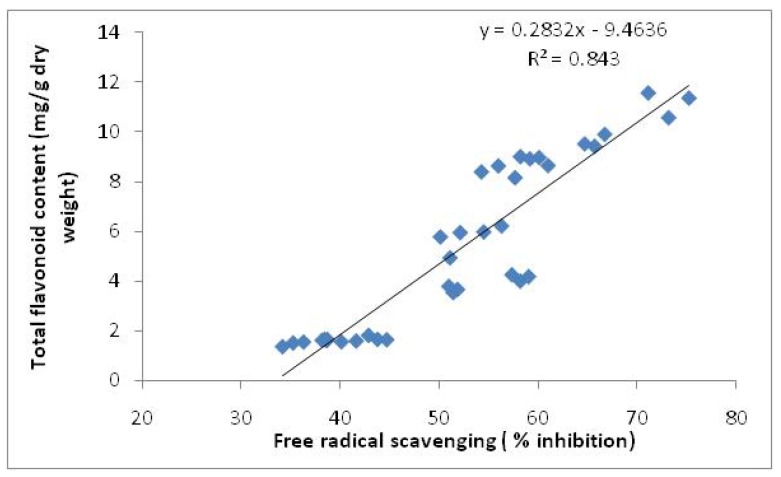
Relationship between total flavonoids and total antioxidant activities of Zingiber officinale.

It was observed that methanolic extracts of the leaves in both varieties had higher activities than those of the rhizomes at different harvest times. The free radical scavenging activities also decreased in the leaves and increased in the rhizomes from 8 to 16 weeks after planting. Leaves of Halia Bara at 8 weeks after planting had higher (73.2%) activities than those of Halia Bentong (65.7%). Similarly, the DPPH radical scavenging activity of the rhizomes of Halia Bara (58.2%) exceeded that of Halia Bentong (51.4%), showing the highest value at 16 weeks after planting. Fifty percent of the free radical scavenging observed in the leaves of both varieties at 8 weeks after planting recorded concentrations of 28 and 39 µg/mL for Halia Bara and Halia Bentong, respectively (Table 4). With increasing growth period, the IC_50_ in the leaves also increased (Table 4). The IC_50_ values of rhizome extracts were only observed at just 16 weeks after planting. A positive relationship between phenolic components and flavonoids with free radical scavenging had been reported in previous studies [9,30,31,32,33]. Since our previous results (Table 2) had shown a decrease in the concentrations of most of the flavonoids *viz.* quersetin, catechin and kaempferol, in the leaves from 8 to 16 weeks after planting, while they increased in the rhizomes during the same time, we could suggest that the decreasing DPPH activities of the leaves from 8 to 16 weeks could be related to the decreasing flavonoid concentrations during this growth period. A positive relationship between total flavonoids and total antioxidant activities of *Zingiber officinale* was also observed in this study (Figure 4).

The increased DPPH activities of rhizomes is related to an increase and accumulation of flavonoids in the rhizomes from 8 till 16 weeks, although the DPPH radical scavenging abilities of the extracts were less than those of BHT (96.21%) and α-tocopherol (89.57%) at 45 µg/mL. The present findings seemed to be consistent with other research. Essential oils extracted from leaves of *Aframomum giganteum* had higher antioxidant activity compared to the rhizomes [34] whilst leaves of *Alpinia zerumbet* (Zingiberaceae) showed higher inhibition of β-carotene oxidation and radical scavenging activity than did rhizomes [35]. Antioxidants are secondary metabolites produced by most of plants but in different content to protect against oxidative damage by free radicals [34,35]. In the family Zingiberaceae and especially *Zingiber officinale*, it is generally believed that antioxidants produced by the plant are transported to the rhizomes where they are accumulated [17,36,37]. This would suggest that the rhizomes should have had higher antioxidant activity than other plant parts, but the results of this study showed that this might not be true as majority of the species studied had significantly higher flavonoids contents and antioxidant activities in the leaves than in rhizomes. Similar observations have been reported by Chan *et al.* [4] and Ghasemzadeh *et al.* [9] who observed that the leaves of ginger with high TF levels also had high antioxidant activities compared to the rhizomes. The antioxidant study showed that the ginger extracts have the good proton-donating ability and could serve as free radical inhibitors or scavengers, acting possibly as primary antioxidants. 

## 3. Experimental 

### 3.1. Plant material

Two varieties of *Zingiber officinale* Roscoe (Halia Bentong and Halia Bara) rhizome seeds were germinated in 10 cm diameter pots containing peat moss for two weeks and then transferred to 15 × 18 cm white polyethylene bags containing a soiless mixture of burnt rice husks and coco peat at a ratio of 1:1. The plants were grown under glasshouse conditions at the Faculty of Agriculture Controlled Environment Complex of University Putra Malaysia (UPM) where the daily mean irradiance was recorded at approximately 790 µmol m^-2^ s^-1^. The plants were harvested at 16 weeks, when the leaves, stems, and rhizomes were separated. Once dried (freeze dry), they were all kept at -80 ºC for future analysis. 

### 3.2. Determination of total flavonoid contents (TF) 

The TF were measured following a previously reported spectrophotometric method [38]. Briefly, extracts of each plant material (1 mL containing 0.1 mg/mL) were diluted with water (4 mL) in a 10 mL volumetric flask. Initially, 5% NaNO_2_ solution (0.3 mL) was added to each volumetric flask; at 5 min, 10% AlCl_3_ (w/w) was added; and at 6 min, 1.0 M NaOH (2 mL) was added. Water (2.4 mL) was then added to the reaction flask and mixed well. Absorbance of the reaction mixture was read at 430 nm. The results were expressed in mg quercetin/g dry weight by comparison with the quercetin standard curve, which was made in the same condition. 

### 3.3. High Performance Liquid Chromatography (HPLC) apparatus 

#### 3.3.1. Extract preparation

Aliquots of leaves and rhizomes (0.25 g) were extracted with 60% aqueous methanol (20 mL). Then 6 M HCl (5 mL) was added to each extract to obtain a 25 mL solution of 1.2 M HCl in 50% aqueous methanol. Extracts were refluxed at 90 ºC for 2 h. Extract aliquots of 500 μL, taken both before and after hydrolysis, were filtered through a 0.45 µm filter [39].

#### 3.3.2. Analysis of flavonoid composition by HPLC

Reversed-phase HPLC was used to assay compositions of flavonoids. The Agilent HPLC system (Tokyo, Japan) used consisted of a Model 1100 pump equipped with a multi-solvent delivery system and a L-7400 ultraviolet (UV) detector. The column type was an Agilent C_18_ 5 µm, 4.0 mm internal diameter × 250 mm. The mobile phase composed of (A) 2% acetic acid (CH_3_COOH) and (B) 0.5% acetic acid-acetonitrile (CH_3_CN),(50:50 v/v), and gradient elution was performed as follows: 0 min, 95:5; 10 min, 90:10; 40 min, 60:40, 55 min, 45:55; 60 min, 20:80; and 65 min, 0:100. The mobile phase was filtered under vacuum through a 0.45 µm membrane filter before use. The flow rate was 1 mL/min. UV absorbance was measured at 280–365 nm. The operating temperature was maintained at room temperature [40]. Identification of the flavonoids was achieved by comparison with retention times of standards, UV spectra and calculation of UV absorbance ratios after co-injection of samples and standards. Commercial standards were purchased from Sigma–Aldrich (USA).

### 3.4. Determination of antioxidant activities

#### 3.4.1. DPPH radical scavenging assay

1,1-Diphenyl-2-picrylhydrazyl (DPPH) was purchased from Sigma–Aldrich (USA). Butylated hydroxytoluene (BHT) and α-tocopherol were purchased from Merck (India). In order to determine the radical scavenging ability, the method reported by Mensor *et al*. [41] was used. Briefly, 0.3 mM alcohol solution of DPPH (1 mL) was added to samples (2.5 mL) containing different concentrations originating from extracts of different parts of ginger varieties. The samples were first kept in a dark place at room temperature and their absorbance was read at 518 nm after 30 min using a spectrophotometer (U-2001, Hitachi Instruments Inc., Tokyo, Japan). The antiradical activity (AA) was determined using the following formula:

AA% = 100 − ((Abs:sample − Abs:empty sample)× 100)/ Abs:control

Empty samples containing 1 mL ethanol + 2.5 mL from various concentrations of ginger extract; control sample containing 1 mL of 0.3 mM DPPH + 2.5 mL ethanol. BHT (butylhydroxytoluene) and α-tocopherol, were used as positive controls.

### 3.5. Statistical analysis

The experimental design was factorial based on randomized complete block design (RCBD) and the results were expressed as mean ± standard deviation of three replicates. Where applicable, the data were subjected to one-way analysis of variance (ANOVA) and differences between factors were determined by Duncan’s Multiple Range test using the Statistical Analysis System (SAS, 1999) and MSTAT-C programme. 

## 4. Conclusions 

Flavonoids constitute an enormous collection of biologically active compounds that are ubiquitous in plants, many of which have been used in traditional Eastern medicine for thousands of years. Advances in analytical techniques such as High Performance Liquid Chromatography (HPLC) allows one to gain insight on their composition, and to study the activity of their components. Ginger has long been used for the treatment of many pathologies, so its use in the medicinal industry could be stimulated in order to develop new pharmaceuticals with particular antioxidant or antimicrobial pharmacological profiles. Herein, we investigated the properties of two Malaysian young ginger (*Zingiber officinale*) varieties (Halia Bentong and Halia Bara), identifying seven important flavonoid components in these two varieties and revealing for the first time the medicinal potential of their leaves.

We have also found that Halia Bara leaves and rhizomes contained more flavonoid components compared to Halia Bentong and that the leaves of both varieties can be used as food flavourings and in traditional medicine due to their good concentrations of flavonoid components. However, in order to produce young ginger rhizomes with high medicinal component quality and flavoring properties, the plant needs to be harvested at 16 weeks, although the use of the leaf flavonoids would be beneficial. Results of leaf analysis showed that the concentration of flavonoids in the leaves decreased during the growth period with subsequent flavonoid increases in the rhizomes. The levels of medicinal components in the leaves were high at 8 weeks after planting. 

## Figures and Tables

**Table 1:** Accumulation and partitioning of total flavonoids in different parts of two varieties of *Zingiber officinale* at different harvest times (weeks after planting).

Variety	Weeks after planting	TF (mg Quercetin eq./g dry weight)	
		Leaves	Rhizomes
Halia Bentong	8	9.59 ± 0.25b	1.48 ± 0.1g
	12	8.38 ± 0.24c	1.59 ± 0.02g
	16	5.54 ± 0.54e	3.66 ± 0.12f
Halia Bara	8	11.14 ± 0.52a	1.65 ± 0.02g
	12	8.82 ± 0.17c	1.71 ± 0.09g
	16	7.05 ± 1.67d	4.14 ± 0.13f

All analyses were mean of triplicate measurements ± standard deviation. Means not sharing a common letter were significantly different at p ≤ 0.05.

**Table 2:** Concentration of flavonoids components in the leaves and rhizomes harvested at 8, 12 and 16 weeks after planting in two varieties of *Zingiber officinale*.

Varieties	Parts	Weeks after planting	Flavonoid components (mg/g dry weight)					
			Q	R	C	EPC	N	K
Halia Bentong	Leaves	8	0.926±0.035bcd	0.152±0.017d	0.41±0.04bc	0.113±0.014c	0.054±0.007a	0.051±0.014abcd
	Leaves	12	0.857±0.03cde	0.46±0.041a	0.35±0.04d	0.104±0.0065d	0.055±0.007a	0.05±0.003abcd
	Leaves	16	0.836±0.0015def	0.054±0.005e	0.31±0.045d	0.092±0.0015e	0.049±0.0015ab	0.043±0.0055cd
	Rhizomes	8	0.505±0.034h	0.226±0.016c	0.20±0.005e	0.049±0.0015h	0.031±0.004e	0.023±0.006e
	Rhizomes	12	0.787±0.002ef	0.282±0.011b	0.24±0.03e	0.058±0.0021g	0.043±0.007bcd	0.042±0.008d
	Rhizomes	16	0.803±0.028def	0.311±0.022b	0.36±0.055cd	0.077±0.002f	0.046±0.006bc	0.045±0.0035cd
Halia Bara	Leaves	8	1.299±0.249a	0.211±0.014c	0.56±0.07a	0.19±0.0031a	0.04±0.003cd	0.054±0.0075abc
	Leaves	12	1.033±0.002b	0.215±0.022c	0.55±0.04a	0.142±0.002b	0.042±0.0055cd	0.044±0.006cd
	Leaves	16	0.978±0.092bc	0.205±0.023c	0.45±0.06b	0.11±0.0031cd	0.039±0.003d	0.048±0.0045bcd
	Rhizomes	8	0.641±0.001g	0.423±0.038a	0.36±0.045cd	0.020±0.0011i	0.015±0.003f	0.048±0.006bcd
	Rhizomes	12	0.713±0.035fg	0.305±0.013b	0.41±0.03bc	0.047±0.002h	0.018±0.0035f	0.057±0.0035ab
	Rhizomes	16	0.865±0.044cde	0.324±0.038b	0.45±0.03b	0.091±0.002e	0.02±0.002f	0.06±0.0045a

Q: quercetin; R: rutin; C: catechin; EPC:epicatechin; N: naringenin; K: kaempferol; All analyses were mean of triplicate measurements ± standard deviation. Means not sharing a common letter were significantly different at p ≤ 0.05.

**Table 3:** DPPH scavenging activities of the methanolic extracts in different parts of two varieties of *Zingiber officinale.* BHT and α-tocopherol were used as positive controls.

Variety	Extraction source	Weeks after planting		
		8	12	16
Halia Bentong	Leaf	65.71±1.01a	55.98±1.69e	51.13±1.06f
	Rhizome	35.28±1.06i	40.16±1.49h	51.43±0.44f
Halia Bara	Leaf	73.16±2.05a	60.08±0.91a	56.34±1.84e
	Rhizome	38.47±0.26h	43.81±0.91g	58.2±0.84d
Control	BHT	96.21±1.87		
	α-tocopherol	89.57±1.74		

Results are expressed in percent of free radical inhibition. All analyses were mean of triplicate measurements ± standard deviation. Means not sharing a common letter were significantly different at p ≤ 0.05.

**Table 4:** IC_50_ values of ginger (*Zingiber officinale*) extracts in the DPPH assay.

Variety	Extraction source	Weeks after planting		
		8	12	16
			IC _50_	
Halia Bentong	Leaf	39	40	42
	Rhizome	no	no	46.5
Halia Bara	Leaf	28	34	35
	Rhizome	no	no	40
Control	BHT	21	21	21
	α-tocopherol	16	16	16

Results expressed in µg/mL of concentration. no: not observed.
